# Supplementary figures and images for: Nano-curcumin enhances the sensitivity of tamoxifen-resistant breast cancer cells via the Cyclin D1-DILA1 axis and the PI3K/AKT/mTOR pathway downregulation
Source: PLoS One. 2025 Dec 5;20(12):e0335165. doi: 10.1371/journal.pone.0335165 (PMC12680210; doi:10.1371/journal.pone.0335165)

S1 Fig

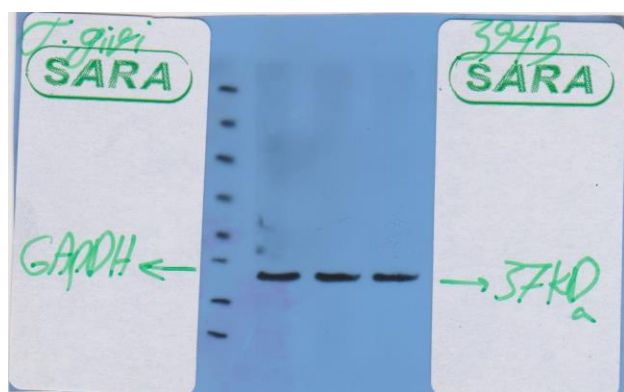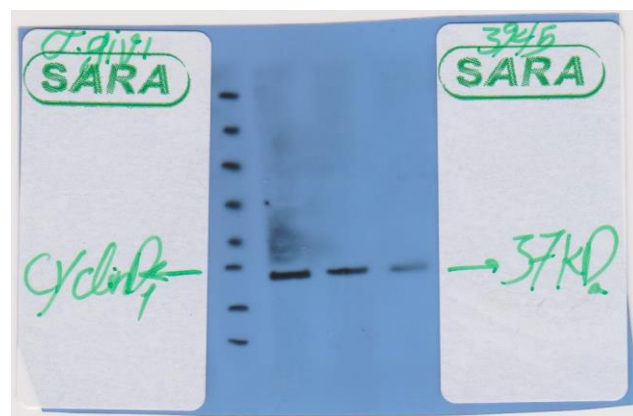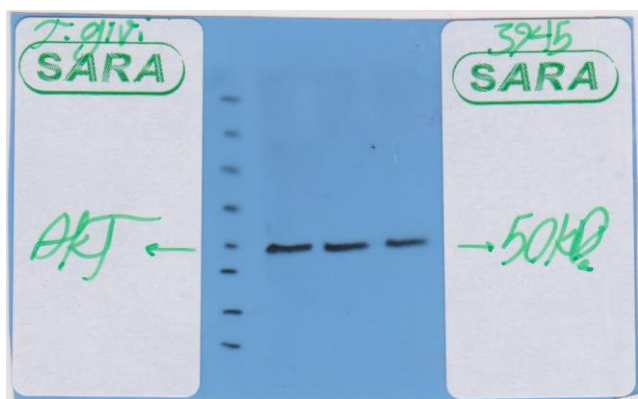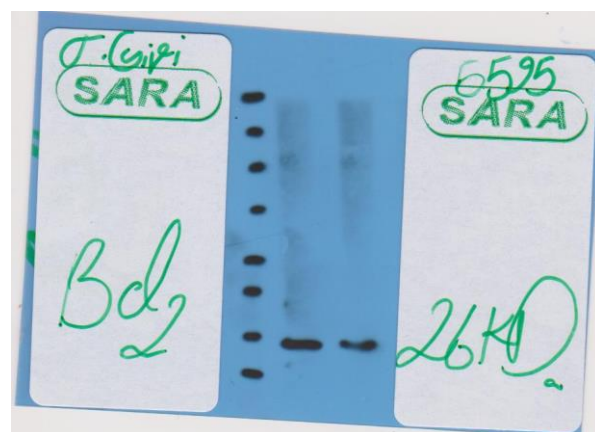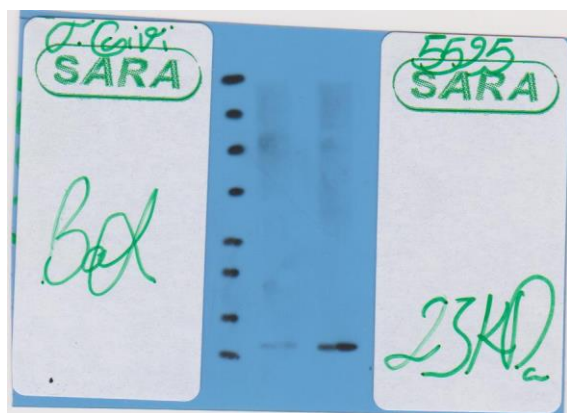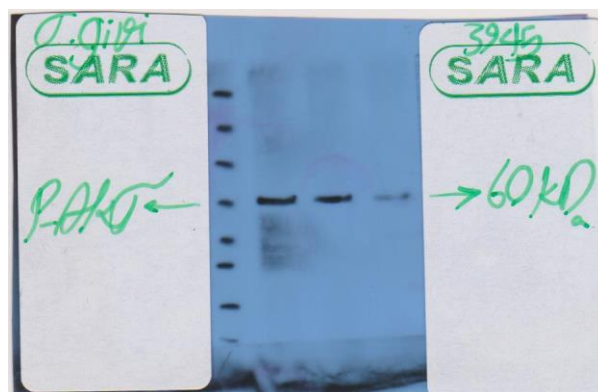

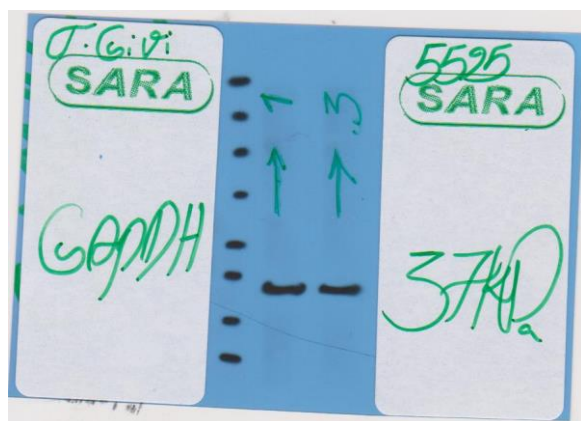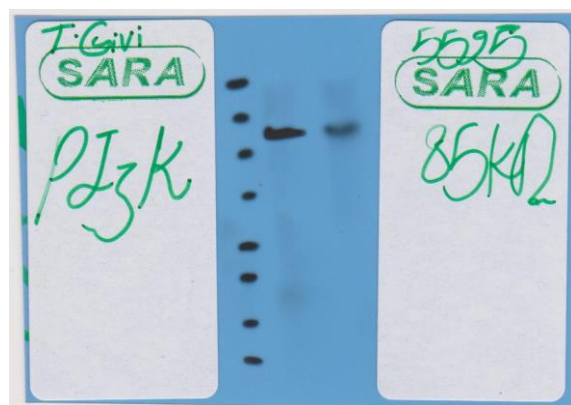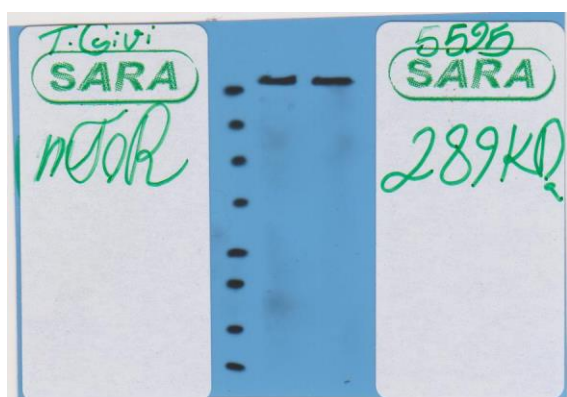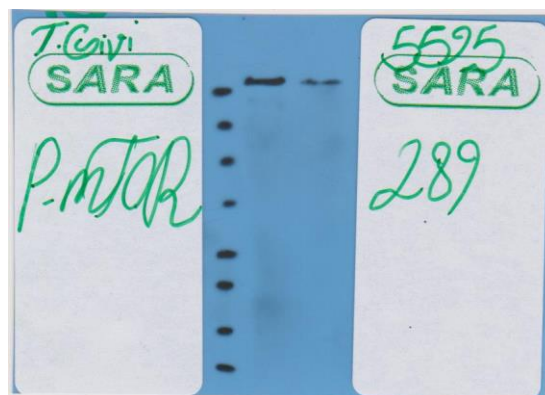

**S1 Fig.** Original Western blot images

Supplement: S1 Fig — (PDF) [file pone.0335165.s001.pdf]
