# Supplementary material for: Nano-curcumin enhances the sensitivity of tamoxifen-resistant breast cancer cells via the Cyclin D1-DILA1 axis and the PI3K/AKT/mTOR pathway downregulation
Source: PLoS One. 2025 Dec 5;20(12):e0335165. doi: 10.1371/journal.pone.0335165 (PMC12680210; doi:10.1371/journal.pone.0335165)
Supplement: S1 Table — (PDF) [file pone.0335165.s002.pdf]

S1 Table

Time	Month 1	Month 2	Month 3	Month 4	Month 5	Month 6
Week 1	0.5 µM	1.5 µM	3.25 µM	5.5 µM	7.5 µM	10 µM
Week 2	0.75 µM	2 µm	3.75 µM	6 µM	8 µM	11 µM
Week 3	1µM	2.25 µm	4.25 µM	6.5 µM	8.5 µM	11.5 µM
Week 4	1.25 µM	3 µM	5 µM	7 µM	9.5 µM	12 µM

S1 Table. The concentration of Tamoxifen was used to induce resistance in MCF7-S for 6 months.
