# Supplementary material for: Nano-curcumin enhances the sensitivity of tamoxifen-resistant breast cancer cells via the Cyclin D1-DILA1 axis and the PI3K/AKT/mTOR pathway downregulation
Source: PLoS One. 2025 Dec 5;20(12):e0335165. doi: 10.1371/journal.pone.0335165 (PMC12680210; doi:10.1371/journal.pone.0335165)
Supplement: S2 Table — (PDF) [file pone.0335165.s003.pdf]

**S2 Table**

| Sequences of primers | | | |
|---|---|---|---|
| Gene | Primer (5'>3') | Size (bp) | Size of PCR product (bp) |
| Forward primer for Cyclin D1 | CCCTCGGTTTCCTACTTCAAA | 21 | 117 |
| Reverse primer for Cyclin D1 | GAAGACCTCCTCCTCGCA | 18 | |
| Forward primer for DILA1 | CCTGTTACAGTGCTGGATTCAT | 22 | 112 |
| Reverse primer for DILA1 | TGTCCTAGATGTCTTCTGGCTT | 22 | |
| Forward primer for GAPDH | TGGAGTCCACTGGCGTCTTC | 20 | 116 |
| Reverse primer for GAPDH | TTGCTGATGATCTTGAGGCTGT | 22 | |
| Forward primer for AKT1 | AACGGGGGCGAGCTGTTC | 18 | 115 |
| Reverse primer for AKT1 | TCTTCTCCGAGTGCAGGTAGTCC | 23 | |
| Forward primer for MTOR | GGGACAGCATGGAAGAATACA | 21 | 109 |
| Reverse primer for MTOR | CAGAGATGTCCAGTCAGCCTG | 21 | |
| Forward primer for PI3K | AGAGGATGATTGCCGCCGTG | 20 | 169 |
| Reverse primer for PI3K | TCTTCTCCTTGTGCAGGTAGTCC | 23 | |
| Forward primer for PTEN | CACACGACGGGAAGACAAGT | 20 | 162 |
| Reverse primer for PTEN | TCCTCTGGTCCTGGTATGAAG | 21 | |
| Forward primer for MMP2 | TCCCAGCGGCCAAAGTTGATC | 21 | 179 |
| Reverse primer for MMP2 | AAGTGGGACAAGAACCAGATC | 21 | |
| Forward primer for RECK | CATCACACAAACTGCCGAGAA | 21 | 96 |
| Reverse primer for RECK | GGCGCAATAATTTTCCACTGCT | 22 | |
| Forward primer for TIMP3 | GTGCAACTTCGTGGAGAGGT | 20 | 109 |
| Reverse primer for TIMP3 | CAGGTAGTAGCAGGACTTGATCTTG | 25 | |
| Forward primer for VEGF | GTGGTGAAGTTCATGGATGTCTA | 23 | |

| | | | |
|---|---|---|---|
| | | | 115 |
| Reverse primer for VEGF | CACAGGATGGCTTGAAGATGTA | 22 | |
| Forward primer for NF-kB | TCTTACACTTAGCAATCATCCAC | 23 | 178 |
| Reverse primer for NF-kB | CCAGCCCTCAGCAAATCCTCC | 21 | |
| Forward primer for BAX | AGAGGATGATTGCCGCCGTG | 20 | 117 |
| Reverse primer for BAX | AGTAGAAAAGGGCGACAACCC | 21 | |
| Forward primer for BCL2 | CCGAGATGTCCAGCCAGCTG | 20 | 119 |
| Reverse primer for BCL2 | CTCAAAGAAGGCCACAATC | 19 | |
| Forward primer for P53 | TCCTCAGCATCTTATCCGAGTG | 22 | 265 |
| Reverse primer for P53 | AGGACAGGCACAAACACGCACC | 22 | |
| Forward primer for CDKN1A (P21) | TCTTGTACCCTTGTGCCTC | 19 | 111 |
| Reverse primer for CDKN1A (P21) | GTGGTAGAAACTCGTCATGC | 20 | |
| Forward primer for CCNA2 (Cyclin A2) | CAATGGATGGTAGTTTTGAGTCAC | 24 | 215 |
| Reverse primer for CCNA2 (Cyclin A2) | TAACCAGTCCACGAGGATAGC | 21 | |
| Forward primer for E-CAD | CGTGTGTGACTGTGAAGGGG | 20 | 124 |
| Reverse primer for E-CAD | GCTCTTTGACCACCGCTCTC | 20 | |
| Forward primer for TWIST | CGCACCCAGTCGCTCAAC | 18 | 174 |
| Reverse primer for TWIST | GGTCATCTTATTGTCCATCGTCG | 23 | |
| Forward primer for c-MYC | CTCCTACGTTGCGGTCACAC | 20 | 163 |
| Reverse primer for c-MYC | CGGGTCGCAGATGAAACTCT | 21 | |

**S2 Table.** The list of primer and oligo sequences was used in this study.
