# Supplementary material for: Nano-curcumin enhances the sensitivity of tamoxifen-resistant breast cancer cells via the Cyclin D1-DILA1 axis and the PI3K/AKT/mTOR pathway downregulation
Source: PLoS One. 2025 Dec 5;20(12):e0335165. doi: 10.1371/journal.pone.0335165 (PMC12680210; doi:10.1371/journal.pone.0335165)
Supplement: Attachment — Submitted filename: tm resistant BC curcumin.pdf [file pone.0335165.s004.pdf]

Comments on “Nano-curcumin enhances the sensitivity of tamoxifen-resistant breast cancer cells via the Cyclin D1-DILA1 axis and P13K/AKT/mTOR pathway”

1. Please mention the type of breast cancer cell line MCF7 is, if ER positive?
2. Please mention the lowest and highest concentration of TAM used over the time course of the assay.
3. Please validate how the TAM sensitivity and resistant cell line was confirmed.
4. In results section, under “Generation of MCF7….” Check the formatting of the paragraph. The second paragraph starts with “and”. Also, Fig1a description is missing. Scale bars need to be added in fig 1D. Please justify the reason behind choosing the gene set in fig1E. For fig 1G, standard deviations, error bars and statistics on biological triplicate are preferred. Quantification of Western blot in fig 1J is needed and please utilize any blank white space in figures. Also, the claim of fold change in terms of protein levels requires validation by stats, which is missing now.
5. Figure 2A and B are dissimilar in terms of headings from fig2C and D. So please be consistent. Also, I would recommend to perform a viability count with Tam in both cells with and without nano-Curcumin as this would particularly have the selection resistance pressure on the TamR cells.
6. Again, the reason for studying gene activity of PTEN gene needs to be specified and delineate on the graphs of fig 3G, H which is sensitive and resistant cells. And western blot quantifications of fig 3I need to be in graph with proper statistics.
7. For fig 4A-C, please confirm if the statistics were done in biological triplicate as the error bars are hardly visible. Besides the quadrant data, a graphical proportion of the population shift of cells is preferred.
8. In Fig5C, for both control and treated cells at 0hr, it seems few cells are already in the wound. So, it is unclear and difficult to measure migration at this state.
9. Lastly the study is only done in MCF cells and any other TNBC cells or ER+ cells are not included, so there is limitation which needs to be stated. But there is scope for future studies.
