# Supplementary material for: Nano-curcumin enhances the sensitivity of tamoxifen-resistant breast cancer cells via the Cyclin D1-DILA1 axis and the PI3K/AKT/mTOR pathway downregulation
Source: PLoS One. 2025 Dec 5;20(12):e0335165. doi: 10.1371/journal.pone.0335165 (PMC12680210; doi:10.1371/journal.pone.0335165)
Supplement: Attachment — Submitted filename: Response to Reviewers2.docx [file pone.0335165.s006.docx]

**Dear Editor-in-Chief and Reviewers**

We sincerely thank the Academic Editor and Reviewers for their constructive and valuable feedback. Their insightful comments helped us to improve the quality and clarity of our manuscript. Below, we provide a point-by-point response. All corresponding changes are included in the revised manuscript and highlighted in the version with track changes.

Reviewer 1

Comment:

I commend the authors for making a sincere effort to address my comments and concerns. However, I believe the overexpression studies involving DILA1 and Cyclin D1 described in Comment 2 will add significant value to this manuscript by further strengthening the mechanism of action of curcumin. As such, I advise the authors to include this data in the manuscript prior to its publication.

We would like to thank the reviewer for their valuable comments on our manuscript. Our laboratory has extensive expertise in investigating the effects of nano-curcumin, and we have published more than 50 well-recognized articles in this field including “ Combination treatment of dendrosomal nanocurcumin and low-level laser therapy develops proliferation and migration of mouse embryonic fibroblasts and alter TGF-β, VEGF, TNF-α and IL-6 expressions involved in wound healing process ” which was published in this journal (1). We explored different pathways and genes involved. The main aim of this article is to demonstrate, in a broader sense, that nano-curcumin can overcome resistance in Er positive breast cancer cell lines. We also had prior experience in performing rescue assays for nano-curcumin effects, which further supports the validity of our experiments(2). Even though, we completely agree that overexpression studies for DILA1 and Cyclin D1 would provide deeper mechanistic insights. However, such experiments require considerable time and resources and are beyond the scope and timeframe of the current study.

To further strengthen the mechanistic basis of our work, we have expanded the Discussion section to:

Emphasize the regulatory role of DILA1 and Cyclin D1 in tamoxifen resistance.

Incorporate supporting data on related molecular pathways.

Highlight preliminary analyses of potential microRNAs that regulate DILA1, which we are actively pursuing in ongoing projects.

We have also clearly stated that overexpression studies will be performed in future investigations to build upon the current findings.

Reviewer 2

Comment:

There are still graphs for cell viability data where some data points the error bar is not visible clear enough. Is that due to very small error - please clarify.

We thank the reviewer for this careful observation. The error bars are included in all graphs, but in some cases, they are very small due to minimal variation across replicates, making them visually difficult to detect.

To address this, we have:

1. Verified and confirmed all statistical analyses.

2. Updated the figure legends to clarify that the error bars are present but too small to be visible in certain plots.

3. Adjusted the formatting of some graphs to improve visibility.

Editor’s Requirements

References

We thank the Editor for the reminder regarding the reference list. Upon review, we identified that Reference 30 had been included in error. This incorrect citation has been removed and the reference list has been updated and re-checked for accuracy and formatting. All in-text citations have been verified and adjusted where necessary to reflect the corrected reference list.

References:

1. Ebrahiminaseri A, Sadeghizadeh M, Moshaii A, Asgaritarghi G, Safari Z. Combination treatment of dendrosomal nanocurcumin and low-level laser therapy develops proliferation and migration of mouse embryonic fibroblasts and alter TGF-β, VEGF, TNF-α and IL-6 expressions involved in wound healing process. PLoS One. 2021 May 6;16(5):e0247098. doi: 10.1371/journal.pone.0247098. PMID: 33956815; PMCID: PMC8101758.
2. Esmatabadi MJD, Motamedrad M, Sadeghizadeh M. Down-regulation of lncRNA, GAS5 decreases chemotherapeutic effect of dendrosomal curcumin (DNC) in breast cancer cells. Phytomedicine. 2018 Mar 15;42:56-65. doi: 10.1016/j.phymed.2018.03.022. Epub 2018 Mar 13. PMID: 29655698.
